# *SLC39A8* missense variant is associated with Crohn's disease but does not have a major impact on gut microbiome composition in healthy subjects

**DOI:** 10.1371/journal.pone.0211328

**Published:** 2019-01-31

**Authors:** Valerie Collij, Floris Imhann, Arnau Vich Vila, Jingyuan Fu, Gerard Dijkstra, Eleonora A. M. Festen, Michiel D. Voskuil, Mark J. Daly, Ramnik J. Xavier, Cisca Wijmenga, Alexandra Zhernakova, Rinse K. Weersma

**Affiliations:** 1 Department of Gastroenterology and Hepatology, University of Groningen, University Medical Center Groningen, RB, Groningen, The Netherlands; 2 Department of Genetics, University of Groningen, University Medical Center Groningen, Groningen, RB, Groningen, The Netherlands; 3 Department of Paediatrics, University of Groningen, University Medical Center Groningen, Groningen, RB, Groningen, The Netherlands; 4 Harvard/MIT Health Science and Technology Institute, Cambridge, Massachusetts, United States of America; Georgia State University, UNITED STATES

## Abstract

**Background:**

Gene-microbiome interactions are important in aetiology and pathogenesis of inflammatory bowel disease, a chronic inflammatory disorder of the gastrointestinal tract consisting of Crohn’s disease and ulcerative colitis. Scarce studies on gene-microbiome interactions show very little overlap in their results. Therefore, it is of utmost importance that gene-microbiome studies are repeated. We aimed to replicate the association between the *SLC39A8* [Thr]391 risk allele and gut microbiome composition in patients with inflammatory bowel disease and healthy controls.

**Methods:**

We collected faecal samples, peripheral blood and extensive phenotype data from 291 patients with inflammatory bowel disease and 476 healthy controls. Carrier status information was obtained from whole exome sequencing data, generated using the Illumina HiSeq. The gut microbiome composition was determined by tag-sequencing the 16S rRNA gene. Associations between carrier status and disease were tested using the Wilcoxon-Mann-Whitney test. Associations between carriers and gut microbiome composition were determined using principal coordinate analyses, variance explained, alpha diversity and additive general linear models in inflammatory bowel disease, healthy controls and all groups combined.

**Results:**

Crohn’s disease patients were more often carriers of the missense variant (21/171, 12.3%) than controls (30/476, 6.3%) (OR = 2.1, P = 0.01). We could not identify associations between carrier status and overall gut microbiome composition and microbial richness in all tested groups after correcting for potential confounding factors. We did identify 37 different operational taxonomical units to be associated with carrier status among the tested groups. Two of these 37 were identified before in the discovery study.

**Conclusions:**

We could confirm the genetic association of the *SLC39A8* [Thr]391 risk allele with Crohn’s disease but we could only limited replicate the association in gut microbiome composition. Independent replication of gene-microbiome studies is warranted to identify true biological mechanisms.

## Introduction

Inflammatory bowel disease (IBD) is a common, chronic disorder of the gastrointestinal tract. Patients with this disease experience periods of inflammation alternated by periods of remission. The most common subgroups of IBD are Crohn’s disease (CD), ulcerative colitis (UC) and inflammatory bowel disease undetermined (IBDU)[1].

In the last ten years, large efforts have been made to identify the genomic landscape of patients with IBD by conducting genome-wide association studies (GWAS). These studies have resulted in the discovery of over 200 genomic regions associated with IBD [2–4]. The genes in these genomic regions indicate that gene-microbe interactions underlie key parts of the pathogenesis of IBD. As a result, studies have now begun to unravel the gut microbiome composition in IBD patients [5,6], and IBD research is moving towards integration of the associated genomic regions with the associated changes in gut microbiome composition.

Recently, three independent studies have reported on the integration of the host genome and gut microbiome composition in the general population in *Nature Genetics* [7–9]. The results of these studies showed very little overlap, probably due to the complexity and variability of the gut microbiome composition. This already starts at the beginning of fecal sampling collection. Differences in collecting methods of these samples or differences in DNA extraction techniques could already lead to differences in results [10]. On top of that, different sequencing techniques were used in these studies; two of them used tag sequencing of the 16S rRNA gene to determine the gut microbiota composition and one study used metagenomics sequencing. Furthermore, over a 100 factors have been identified to be of influence of the gut microbiome composition [11]. In order to make different studies comparable to one another, the same factors should be taken into account. Thus, genome-microbiome associations should be assessed with caution and replication of gene-microbiome associations is certainly warranted [12].

In 2016, Li *et al* reported the identification of a novel exonic missense variant in the *SLC39A8* gene (alanine 391 threonine, rs13107325) that was associated with Crohn’s disease (CD) [13]. Importantly, they also reported that the *SLC39A8* [Thr]391 risk variant for CD was associated with the gut microbiome composition in patients with CD and in healthy controls (HC), using microbiome data of 338 mucosal lavage samples from 171 individuals (including patients with CD and HC) [13].

Given the importance of verifying gene-microbiome associations, we aimed to replicate Li *et al*’s association between the *SLC39A8* [Thr]391 risk allele and gut microbiome composition in faecal samples in our Dutch cohort of patients with IBD and HC which is 4.5 times larger than the original cohort [5,13]. Despite the differences in sample collection methods (lavage vs stool samples) we hypothesize that the influence of the *SLC39A8* missense variant is also present in our faecal samples, because of the large effect of this missense variant on the gut microbiome composition identified earlier by Li *et al* [13].

## Materials and methods

### Cohorts

We included 767 individuals, comprising 171 patients with CD, 104 patients with UC and 16 patients with IBDU from the University Medical Center Groningen (UMCG) IBD cohort and 476 HC from the LifeLines DEEP general population cohort in the Netherlands [5,14]. Faecal samples, peripheral blood, and extensive phenotype data, including age, sex, BMI, and current medication use, were available for all participants. The Institutional Review Board of the University Medical Center Groningen approved both the IBD cohort and the LifelinesDEEP cohort (Institutional Review Board number 2008.338 for the IBD cohort and document number M12.113965 for the LifeLinesDEEP cohort) [5,14].

### Genotyping

The determination of the presence of the *SLC39A8* missense variant was obtained by using whole exome sequencing (WES). For each participant, peripheral blood samples stored in EDTA tubes (BD Vacutainer) were available. DNA extraction was performed using the Qjagon Autopure LS with Puregene chemistry (Qiagen NV, Venlo, Netherlands). Sample preparation was done by using Illumina Nextera prep kit and enrichment of the exonic sequences was performed by hybrid capture using Illumina rapid Capture Enrichment (37 mb target). The illumina hiSeq platform with 150 bp paired reads was used for sequencing. The mapping of reads to the human genome reference sequence (GRCh37) was performed by using BWA-MEM. An average sequencing depth of 20x covering 80 percent was used in the sequencing of all samples. The average of our obtained reads is 90,655,419 (range 51,590,508–201,639,082) for all the samples. Around 94 percent of the targeted regions were covered by 10 times or more and in the case of 20 times or more this percentage is 87 percent. An extensive quality control and variant calling process has been performed on all samples and is described in a previously published study [15].

### Gut microbiome composition

The gut microbiome composition of stool samples was determined using tag-sequencing of the 16S rRNA gene as described previously [5,14]. In short: participants were asked to produce and freeze a stool sample within 15 minutes after production. The samples were collected from the patients’ homes on dry ice and stored in our –80°C freezer. Faecal DNA was isolated by making aliquots and for the isolation of microbial DNA the Qiagen AllPrep DNA/RNA Mini Kit cat #80204 was used. Illumina MiSeq paired-end sequencing of the V4 region of the 16S rRNA gene was performed. The forward primer 515F [*GTGCCAGCMGCCGCGGTAA]* and the reverse primer 806R *[GGACTACHVGGGTWTCTAAT]* was used for this step. The microbiome taxonomy was determined by operational taxonomic unit (OTU) picking using QIIME and Usearch (V.7.0.1090) based on similarity of 97%, and Greengenes (V.13.8) was used as a reference database [5]. This led to the identification of 12,556 OTUs. Samples with less than 10,000 counts were removed. In comparison with the discovery paper, the OTUs were filtered based on availability in at least 10 percent of the samples. Lastly, these OTUs were classified into 250 different taxa by summing the read counts and transform them into relative abundances. The codes used for these analyses are publicly available at:

https://github.com/WeersmaLabIBD/Microbiome/blob/master/16s_qiime2_pipeline.md

### Overall gut microbiome composition and microbial richness

All statistical analyses were performed in each phenotypic cohort (CD, UC and HC) and all groups combined (all phenotypic cohorts including patients with IBDU). The Wilcoxon-Mann-Whitney test was used to identify the possible association between the presence of the *SLC39A8 [Thr]391* risk allele and disease status and the odds ratio was used to determine the quantity of this effect. Overall microbiome composition (beta diversity) was assessed by Bray-Curtis, Jensen-Shannon, Jaccard unweighted Unifrac and weighted Unifrac measurements. Associations between the *SLC39A8 [Thr]391* risk allele and the overall composition of the gut microbiome were estimated by calculating the proportion of variance explained by the mutation on the different beta diversity distance matrix, using a PERMANOVA test as implemented in the *adonis* function of the *vegan* package in R [16]. Additionally, we analysed the alpha diversity by calculating the Shannon, Chao1 and Simpson index as well as the number of observed species per sample using *vegan* and *phyloseq* R packages. Differences in alpha diversity between carriers and non-carriers were analysed through the non-parametric Wilcoxon-Mann-Whitney test. All associations were evaluated with and without taking into account the microbiome confounding factors: age, sex, sequencing depth and for the disease cohort also the disease duration (measured as: years having IBD = fecal sample date—first diagnosis date). For all these tests, p-values of <0.05 were considered statistically significant. The codes are available at:

https://github.com/WeersmaLabIBD/Microbiome/blob/2df040c67c8b75e514abe896def1672bdcb2dee0/SLC39A8_16S

### Association to taxa and OTUs

Possible associations between the individual operational taxonomical units (OTUs) and the *SLC39A8 [Thr]391* risk allele status were determined in the described groups by using univariate and multivariate additive general linear models in the software tool MaAsLin as described previously [5]. Covariates in the multivariate models comprised 11 confounding factors known to influence the gut microbiome composition: age, sex, body mass index, proton-pump inhibitor use, as well as antibiotic use and IBD medication (mesalazines, steroids, thiopurines, methotrexate and TNF-α inhibitors) [5]. For patients with CD and UC, the amount of years having the diagnosis IBD was also added as a covariate in the linear models. All analyses were corrected for multiple testing by using the false discovery rate (FDR, Benjamini Hochberg method) incorporated in the Q-package in R. An FDR of <0.05 was considered statistically significant. In the discovery papers, only analyses in individual OTUs correcting for less confounding factors was performed [13]. In this study, we corrected for more factors, to prevent identifying false-positives by the influence of confounding factors.

## Results

### Clinical characteristics and genetic association

We identified 21 carriers of the *SLC39A8* missense variant in patients with CD (12.3%), 7 carriers in patients with UC (6.7%), 1 carrier in patients with IBDU (6.3%) and 30 carriers in HC (6.3%). Because only 1 patient with IBDU was carrier of the missense variant, the differences in the clinical characteristics between carriers and non-carriers of the *SLC39A8* [Thr]391 risk allele was not assessed in this group as depicted in Table 1. Smoking is defined as current smoker at time of faecal sampling. Active disease is defined as a score of higher than 4 of the Harvey-Bradshaw index (disease severity measure for CD) or a score higher than 2.5 of the Simple Clinical Colitis Activity Index (disease severity measure for UC). There were no statistically significant differences between carriers and non-carriers of the *SLC39A8* missense variant and the clinical characteristics in the described groups (Table 1). We did identify a trend in which carriers of the missense variant have had longer the diagnosis IBD at time of faecal sampling than non-carriers in patients with CD (means of 15 vs 12 years, P = 0.066, as depicted in Table 1). Therefore, we took this factor into account in the analyses of the gene-microbiome interaction. Patients with CD were more often carriers of the *SLC39A8* risk allele than HC (OR = 2.1, P = 0.01). This was not the case for patients with UC, nor for patients with IBDU compared to HC (P = 0.8271 and P = 0.9949 respectively).

**Table 1:** Clinical characteristics of patients with CD, UC and healthy controls.

	Crohn’s disease (n = 171)			Ulcerative colitis (n = 104)			Healthy controls (n = 476)		
Factors	*SLC39A8 risk allele carrier*	*No SLC39A8 risk allele carrier*	*P-Value*	*SLC39A8 risk allele carrier*	*No SLC39A8 risk allele carrier*	*P-Value*	*SLC39A8 risk allele carrier*	*No SLC39A8 risk allele carrier*	*P-Value*
**Number**	21	150	NA	7	97	NA	30	446	NA
**Age in years (SD)**	42.1 (15.3)	41.0 (14.1)	0.7047	55 (19.0)	46.7 (14.3)	0.159	44.4 (13.0)	45.8 (13.5)	0.6157
**Males (%)**	6 (28.6)	51 (34.0)	0.8048	3 (42.9)	47 (48.5)	1	12 (40)	211 (47.3)	0.5568
**BMI (SD)**	25.7 (5.1)	24.7 (4.7)	0.5267	26.5 (5.2)	26.5 (4.4)	0.9534	24.4 (4.0)	24.9 (3.8)	0.4268
**PPI (%)**	8 (38.1)	31 (20.1)	0.09497	2 (28.6)	11 (11.3)	0.2111	1 (3.3)	19 (4.3)	1
**Antibiotics (%)**	6 (28.6)	33 (22.0)	0.5787	2 (28.6)	13 (13.4)	0.2653	0 (0)	0 (0)	1
**Mesalazines (%)**	0 (0)	12 (8.0)	0.3651	6 (85.7)	76 (78.4)	1	0 (0)	0 (0)	1
**Steroids (%)**	6 (28.6)	29 (19.3)	0.3853	1 (14.3)	23 (23.7)	1	0 (0)	0 (0)	1
**Thiopurines (%)**	7 (33.3)	50 (33.3)	1	2 (28.6)	29 (29.9)	1	0 (0)	0 (0)	1
**Methotrexate (%)**	3 (14.3)	19 (12.7)	0.7369	0 (0)	1 (1.0)	1	0 (0)	0 (0)	1
**Anti-TNFα (%)**	9 (42.9)	64 (42.7)	1	0 (0)	10 (10.3)	1	0 (0)	0 (0)	1
**CRP mean (range)**	2.2 (1–14)	1.8 (1–13)	0.3223	1.4 (1–3)	1.4 (1–8)	0.7179	NA	NA	NA
**Fcal mean (range)**	12 (1–67)	9 (1–89)	0.665	14 (1–62)	15 (1–390)	0.7141	NA	NA	NA
**Active disease (%)**	8 (38)	31 (22)	0.1875	2 (29)	25 (26)	1	NA	NA	NA
**Disease location**									
***Ileum***	9 (43)	51 (38)	0.7163	0 (0)	0 (0)	1	NA	NA	NA
***Colon***	5 (24)	25 (19)	0.6782	7 (100)	97 (100)	1	NA	NA	NA
***Both***	7 (33)	59 (44)	0.6859	0 (0)	0 (0)	1	NA	NA	NA
**Smoking (%)**	8 (38)	43 (30)	0.5916	2 (29)	13 (13.4)	0.5849	14 (24)	140 (20)	0.618
**Disease duration mean (range)**	15 (1–29)	12 (1–48)	0.066	10 (2–17)	11 (1–37)	0.8301	NA	NA	NA

SD standard deviation; BMI body mass index; PPI proton pump inhibitors; Anti-TNFα tumour-necrosis-factor-α inhibitors; CRP C-reactive protein; Fcal Fecal calprotectin; NA not applicable.

### Overall gut microbiome composition and microbial richness

**Fig 1:**
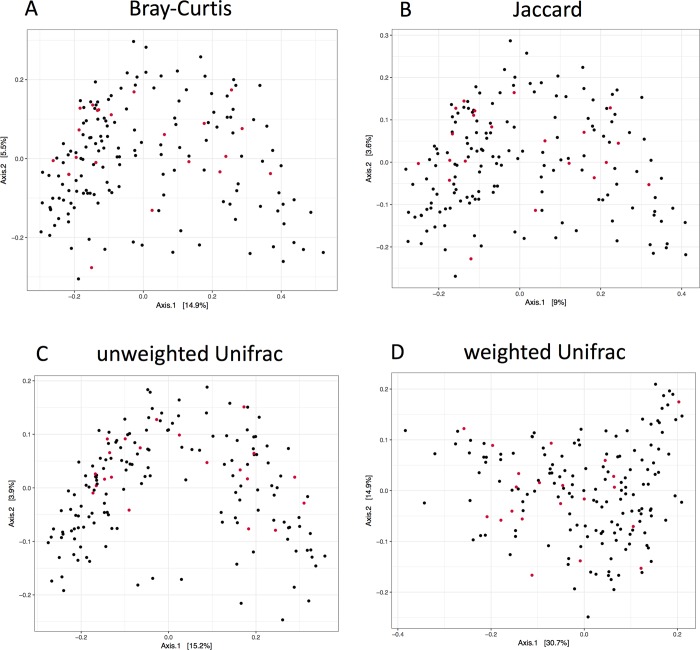
Beta diversity within Crohn’s disease by using four methods. Principal coordinate analysis of gut microbiome composition generated using 16S rRNA sequencing of stool samples of 171 patients with CD. Depicted are four different methods to identify the beta diversity of these samples: A) Bray-Curtis distances, B) Jaccard, C) unweighted Unifrac and D) weighted Unifrac. The 21 *SLC39A8 [Thr]391* risk carriers are shown by red dots and 150 non-carriers by black dots. There was no statistically significant association between the *SLC39A8 [Thr]391* risk allele and beta diversity identified in CD, nor in the different methods used.

**Fig 2:**
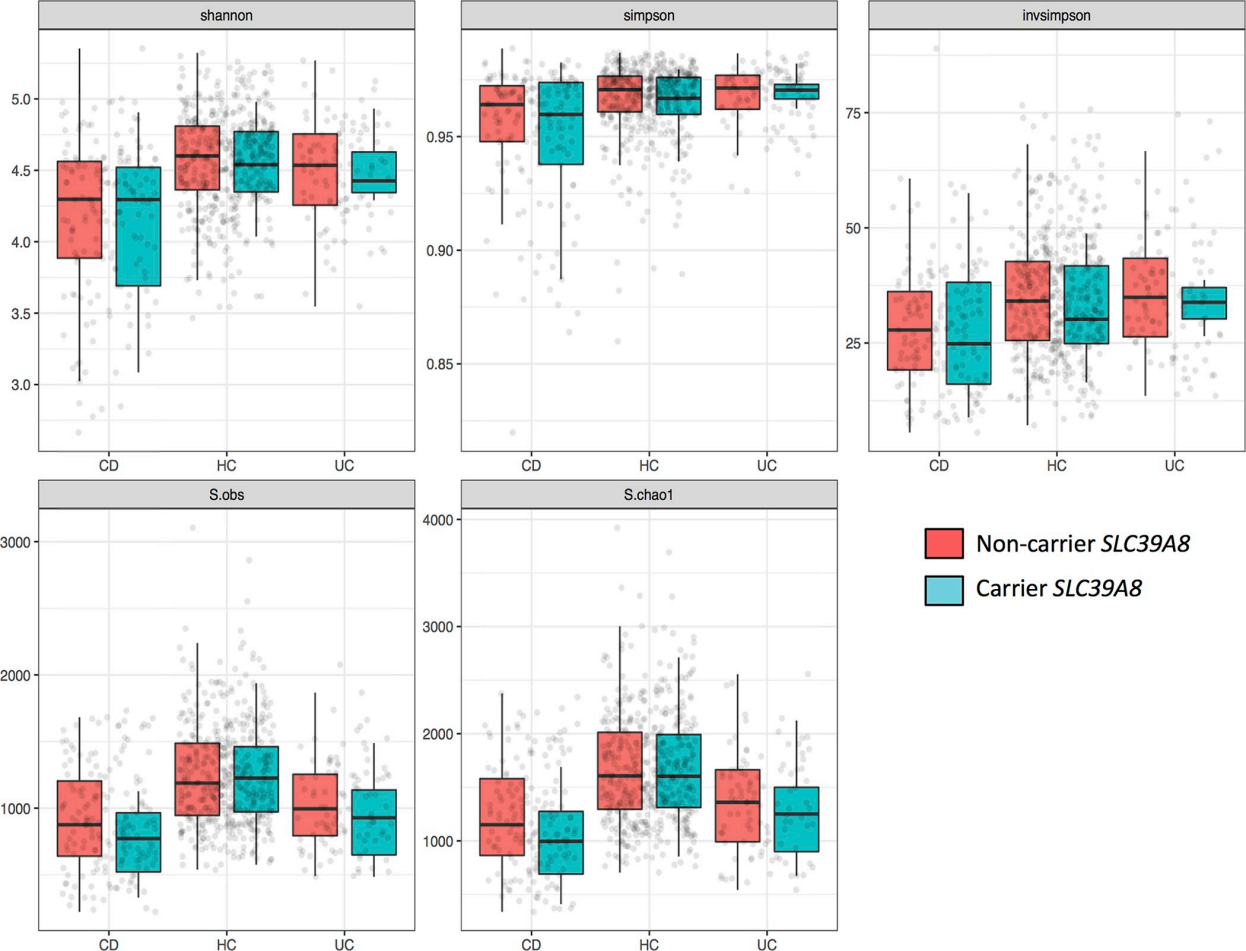
Alpha diversity within CD, UC and HC by using five methods. Alpha diversity calculated by five different methods, from left to right: Shannon Index, Simpson, inversed Simpson, observed species and Chao1. Carrier status does not show statistically significant differences in non-carriers and carriers of the *SLC39A8* missense variants in Crohn’s disease, healthy controls and ulcerative colitis.

In overall gut microbiome composition, none of the tested groups showed any differential clustering or distribution between mutation and non-mutation carriers in the principal coordinate analyses of beta diversity (Fig 1 for within CD, S1–S3 Figs for within UC, HC and all groups combined). We did identify a statistically significant difference between carrier status and beta diversity in all groups combined in the unweighted Unifrac calculations of beta diversity, without correcting for any potentially confounding factors (P = 0.024, S1 Table). Since the missense variant is enriched in CD, we also added diagnosis in the corrected analysis of beta diversity and carrier status. After correction, carrier status was not statistically significant anymore on beta-diversity in all groups combined using unweighted Unifrac (P = 0.464, S2 Table). Furthermore, we could not identify any statistically significant changes between carrier status of the missense variant and alpha diversity in all tested groups, both in corrected and uncorrected analyses (Fig 2 for within CD, UC and HC, S4 Fig for all groups combined and S3 Table).

### Associations individual OTUs and taxa

We were able to identify associations between carrier status of the *SLC39A8* missense variant and individual OTUs; 2 OTUs in the univariate analyses and 37 in the multivariate analyses (S4 Table). In the univariate analyses we identified in both CD and UC 1 associated OTU (OTU-IDs = 2210025 and 4374663, FDR = 0.002 and 0.01 respectively) and none in HC or all groups combined. In the multivariate analyses we identified 5 OTUs in CD, 5 in UC, 14 in HC and 14 in all groups combined (S4 Table). For example, the family Lachnospiraceae was statistically significantly increased in all tested groups in *SLC39A8* missense variant carriers compared to non-carriers, represented by 10 different OTUs divided over the tested groups. Only 2 of these 10 OTUs (OTU-ID 329703 and 4343184, FDR = 0.02 and 0.03, respectively), were also statistically significantly associated with carrier status in non-overweight healthy controls in the discovery paper [13]. Another example is in patients with UC, HC and all groups combined, in which the family Ruminococcaceae was increased in carriers compared to non-carriers. Ruminococcaceae was represented in 12 different OTUs divided over the mentioned phenotypic cohorts, in which 1 OTU (OTU-ID 195950, FDR = 0.048) showed overlap with the findings of the discovery paper^13^. From the 877 associated OTUs from the discovery paper [13], we were only able to identify 5 of them to be statistically significantly associated with carrier status of the *SLC39A8* missense variant in our cohort (S4 Table). However, we could not identify any statistically significant associations between the *SLC39A8* [Thr]391 risk allele and the individual microbial taxa in the tested groups, in either the univariate- or multivariate analysis in which we corrected for covariates.

## Discussion

The aim of this study was to replicate the finding of Li et al’s association between the *SLC39A8 [Thr]391* risk allele and gut microbiome composition in our independent cohort of patients with CD, UC, IBDU and HC, which was 4.5 times larger than the original cohort. This provides an increased power in order to detect true associations between carriers and microbial changes. The *SLC39A8* gene is known as a transporter of Zinc [17]. Zinc deficiency has been associated before with a boost of inflammatory responses and with the increase of oxidative stress, indicating the role of Zinc in immune functions [18,19]. The role of Zinc has also been studied in the context of IBD, in which an in vitro study has shown that Zinc affects the integrity of the intestinal mucosa [20]. In our previous study we have shown multiple factors to be associated with the gut microbiome composition in the context of IBD [5]. Given the large effects of the missense variant on the gut microbiome composition observed by the discovery paper, the association of the missense variant to Crohn’s disease and the role of Zinc on the immune system, we hypothesized that this missense variant could also be of influence in the altered gut microbiome composition in IBD we observed earlier [5,13,17–20].

In this study we could identify the genetic variant to be associated with CD. In addition, the impact on the microbiome composition was limited to a few OTUs, which due differences between boths studies could not be directly compared. However, in OTU identification, we could only replicate 5 OTUs to be associated with carrier status from the discovery paper [13]. When we restrict our analysis to taxonomical level, we could not identify any associations.

Microbial richness and overall gut microbiome composition changes previously reported, could not be replicated. Although the mutation carrier status was statistically significantly associated with beta diversity in the uncorrected analysis of the group all combined by using unweighted Unifrac, after correction for IBD diagnosis, this was not statistically significant anymore. Since the missense variant is enriched in CD, the difference was most likely to be explained by the diagnosis IBD, instead of carrier status of the missense variant. This highlights the importance of considering other factors when performing association studies in IBD context.

We have observed in our results that all identified OTUs were characterized by low mean read counts, ranging from 0–125 (S4 Table). This is also observed in the discovery paper, in which 85% of their identified OTUs also had mean read counts ranging between these values [13]. Therefore, it is very hard to determine if the reported results are indeed positive findings or false positive results. It seems that on top of filtering the minimum prevalence of each taxon (done at 10% in discovery and replication cohort), filtering for minimum abundance of each OTU could reduce the amount of variation between cohorts but also reduce the amount of potentially false positive associations. However, the observed differences between our study and the discovery paper could also be due to the different experimental design [13]. The discovery paper used mucosal lavage samples while the presented study uses faecal content. Although both methods target the same ecosystem, the gut microbiota, different collection methods as well as the use of intestinal preparation for the mucosal lavage can introduce significant changes in the proportion of microbes characterized [21]. In addition, we should also consider the possibility that the findings are cohort specific due to geographical and cultural differences. Furthermore, some extra variation could be introduced by discrepancies in the computational processing and analyses of the samples. Finally, the effect of individual OTUs could be biologically that small, that these effects cannot be detected in alpha and beta diversity.

In the discovery paper of Li *et al*, large effects of the *SLC39A8* missense variant on gut microbiome composition has been described, indicated by the large numbers of associations and effect sizes identified (more than 800 individual OTUs) [13]. In the past, strong signals on the gut microbiota has been replicated, despite different methodologies used. One example of these associations are the one between CD and the increased abundance of the family Enterobacteriaceae. This association has been identified in both adults and paediatric patients with CD, in different sample types (faecal samples and gut mucosal biopsies) and in multiple disease locations [22–28]. On top of that, our sample size is 4.5 times as large as the discovery study. Taken these considerations into account, we hypothesized that despite different sampling collection methods used, we would expect that the positive gene-microbiome signal could also be reproduced in our faecal samples.

Since IBD research is still in the early phase of discovering genome-microbiome associations, the lack of replication is not uncommon in IBD cohorts or in general population studies. Previous studies on IBD patients, in which functional variants in the mucus layer gene *FUT2*, the bacterial antigen receptor gene *NOD2*, and the autophagy gene *ATG16L1* were associated with the gut microbiome [25,29,30], could also not be replicated [5]. Nor could the interactions between the variant in *NOD2* or the variant in the vitamin D receptor (VDR) and the gut microbiome in the general population be replicated [7–9].

Genome-microbiome associations are hard to discover, since the presumed effect of individual genomic variants on the gut microbiome is small, whereas the effect of environmental factors on the gut microbiome can be much larger. Recently, we reported that the variance of the gut microbiome is partly explained by over 100 phenotypes and environmental factors, including medication use and diet [11]. In addition, correcting for multiple testing in genome-wide, microbiome-wide association studies is complex, since the number of tests is hundreds of times larger than in GWAS because of the addition of hundreds more microbial features.

## Conclusions

Therefore, we argue that in future gene-microbiome studies much larger sample sizes, more stringent statistical analyses (especially with regard to mean counts of OTUs and correcting for confounding factors), replication in independent cohorts and elaborate descriptions of the methods used are needed to pinpoint genome-microbiome associations in both IBD and HC.

## Supporting information

S1 Fig Beta diversity within ulcerative colitis by using four methods. Principal coordinate analysis of gut microbiome composition generated using 16S rRNA sequencing of stool samples of 104 patients with UC. Depicted are four different methods to identify the beta diversity of these samples: A) Bray-Curtis distances, B) Jaccard, C) unweighted Unifrac and D) weighted Unifrac. The 7 *SLC39A8 [Thr]391* risk carriers are shown by red dots and 97 non-carriers by black dots. There was no statistically significant association between the *SLC39A8 [Thr]391* risk allele and beta diversity identified in UC, nor in the different methods used.
(TIFF)

S2 Fig Beta diversity within healthy controls by using four methods. Principal coordinate analysis of gut microbiome composition generated using 16S rRNA sequencing of stool samples of 476 healthy controls. Depicted are four different methods to identify the beta diversity of these samples: A) Bray-Curtis distances, B) Jaccard, C) unweighted Unifrac and D) weighted Unifrac. The 30 *SLC39A8 [Thr]391* risk carriers are shown by red dots and 446 non-carriers by black dots. There was no statistically significant association between the *SLC39A8 [Thr]391* risk allele and beta diversity identified in HC, nor in the different methods used.
(TIFF)

S3 Fig Beta diversity in all groups combined by using four methods. Principal coordinate analysis of gut microbiome composition generated using 16S rRNA sequencing of stool samples of all 767 participants. Depicted are four different methods to identify the beta diversity of these samples: A) Bray-Curtis distances, B) Jaccard, C) unweighted Unifrac and D) weighted Unifrac. The 59 *SLC39A8 [Thr]391* risk carriers are shown by red dots and 708 non-carriers by black dots. After correction, there was no statistically significant association between the *SLC39A8 [Thr]391* risk allele and beta diversity identified in all groups combined, nor in the different methods used.
(TIFF)

S4 Fig Alpha diversity in all groups combined by using five methods. Alpha diversity calculated by five different methods, from left to right: Shannon Index, Simpson, inversed Simpson, observed species and Chao1. Carrier status does not show statistically significant differences in non-carriers and carriers of the *SLC39A8* missense variants in all groups combined.
(TIFF)

S1 Table Uncorrected analyses of variance explained by carrier status in beta diversity by using Jenson-Shannon, Jaccard, Bray-Curtis, unweighted Unifrac and weighted Unifrac. These analyses were performed in all tested groups: Patients with CD, patients with UC, HC and all combined. A statistically significant difference was only identified between carrier status and beta diversity in all groups combined by using the method unweighted Unifrac without correction of disease status. In patients with CD and UC these analyses were also performed in patients with a BMI < 25.
(XLSX)

S2 Table Corrected analyses of variance explained by carrier status and beta diversity by using Jenson-Shannon, Jaccard, Bray-Curtis, unweighted Unifrac and weighted Unifrac. These analyses were performed in all tested groups: Patients with CD, patients with UC, healthy controls and all combined. In patients with CD and UC, also disease duration was added in the analyses. By correcting for different factors, carrier status was not associated to changes in beta diversity in all tested groups. This was also the case for all different methods used.
(XLSX)

S3 Table P-values of carrier status and alpha diversity. Carrier status was not associated to changes in alpha diversity. This was the case for all tested groups: Patients with CD, patients with UC, HC and all combined. Also the different methods used for calculating alpha diversity, and correcting or not correcting for potential confounding factors led to the same result.
(XLSX)

S4 Table Univariate and multivariate analyses between specific OTUs and carrier status (FDR < 0.05), red = increased abundance, blue = decreased abundance. A total of 2 OTUs in the univariate and 37 OTUs in the multivariate analyses were identified to be associated to the *SLC39A8* missense variant and individual OTUs. The asterisk indicates OTUs which have also been identified in the discovery paper.
(XLSX)
