# Supplementary figures and images for: SLC39A8 missense variant is associated with Crohn's disease but does not have a major impact on gut microbiome composition in healthy subjects
Source: PLoS One. 2019 Jan 31;14(1):e0211328. doi: 10.1371/journal.pone.0211328 (PMC6354981; doi:10.1371/journal.pone.0211328)

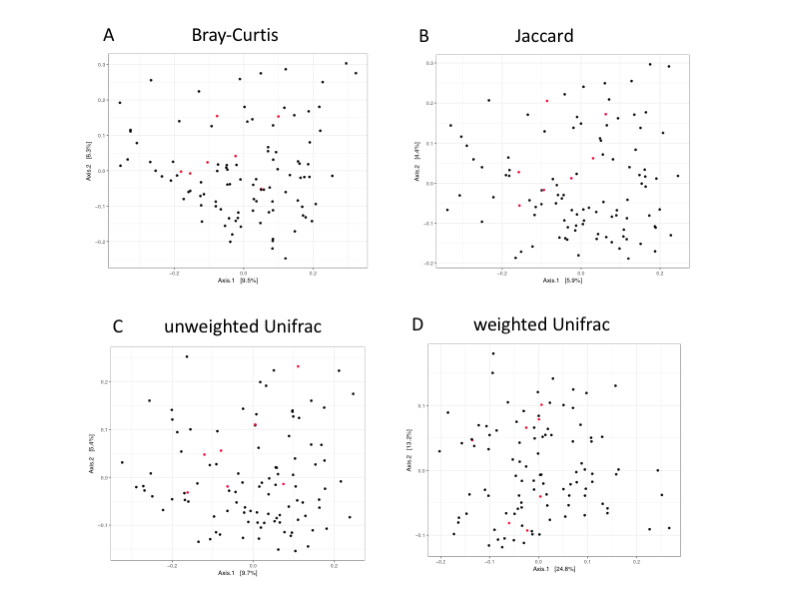

Supplement: S1 Fig — Principal coordinate analysis of gut microbiome composition generated using 16S rRNA sequencing of stool samples of 104 patients with UC. Depicted are four different methods to identify the beta diversity of these samples: A) Bray-Curtis distances, B) Jaccard, C) unweighted Unifrac and D) weighted Unifrac. The 7 SLC39A8 [Thr]391 risk carriers are shown by red dots and 97 non-carriers by black dots. There was no statistically significant association between the SLC39A8 [Thr]391 risk allele and beta diversity identified in UC, nor in the different methods used. (TIFF) [file pone.0211328.s001.tiff]

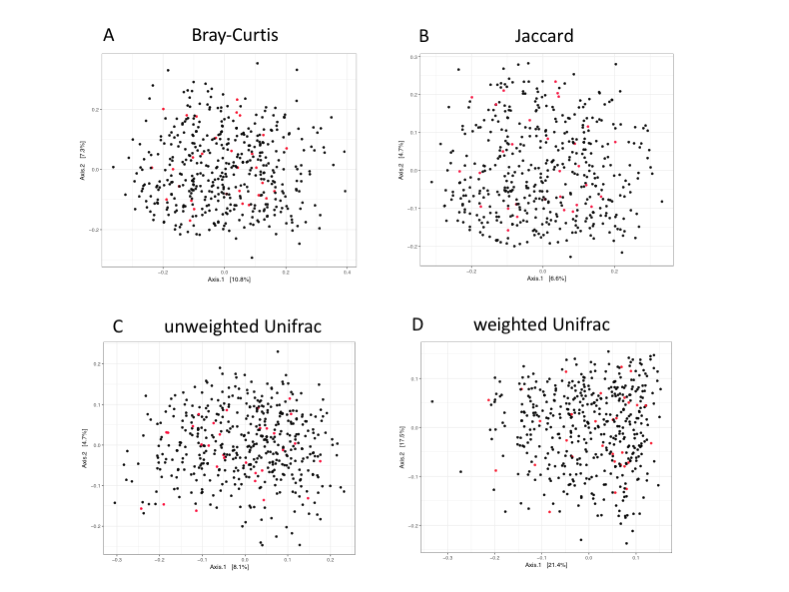

Supplement: S2 Fig — Principal coordinate analysis of gut microbiome composition generated using 16S rRNA sequencing of stool samples of 476 healthy controls. Depicted are four different methods to identify the beta diversity of these samples: A) Bray-Curtis distances, B) Jaccard, C) unweighted Unifrac and D) weighted Unifrac. The 30 SLC39A8 [Thr]391 risk carriers are shown by red dots and 446 non-carriers by black dots. There was no statistically significant association between the SLC39A8 [Thr]391 risk allele and beta diversity identified in HC, nor in the different methods used. (TIFF) [file pone.0211328.s002.tiff]

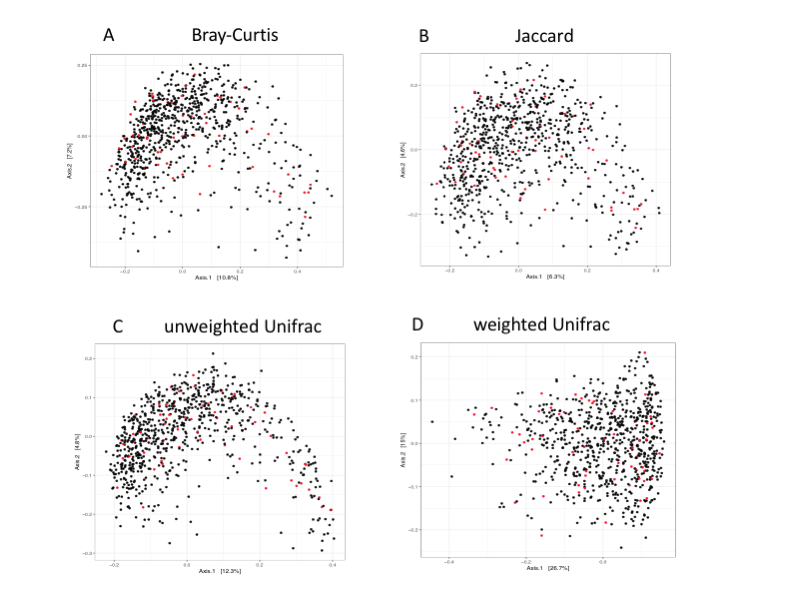

Supplement: S3 Fig — Principal coordinate analysis of gut microbiome composition generated using 16S rRNA sequencing of stool samples of all 767 participants. Depicted are four different methods to identify the beta diversity of these samples: A) Bray-Curtis distances, B) Jaccard, C) unweighted Unifrac and D) weighted Unifrac. The 59 SLC39A8 [Thr]391 risk carriers are shown by red dots and 708 non-carriers by black dots. After correction, there was no statistically significant association between the SLC39A8 [Thr]391 risk allele and beta diversity identified in all groups combined, nor in the different methods used. (TIFF) [file pone.0211328.s003.tiff]

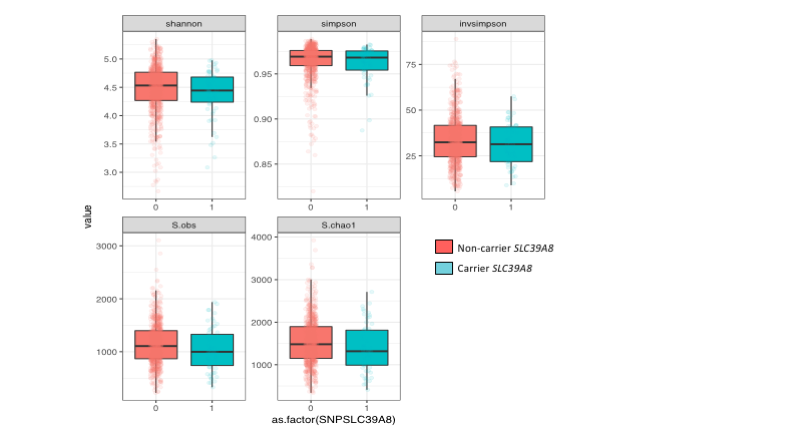

Supplement: S4 Fig — Alpha diversity calculated by five different methods, from left to right: Shannon Index, Simpson, inversed Simpson, observed species and Chao1. Carrier status does not show statistically significant differences in non-carriers and carriers of the SLC39A8 missense variants in all groups combined. (TIFF) [file pone.0211328.s004.tiff]
